# Association of prenatal substance exposure and the development of the amygdala, hippocampus, and parahippocampus

**DOI:** 10.1515/jom-2023-0277

**Published:** 2024-06-26

**Authors:** Micah Hartwell, Molly Bloom, Covenant Elenwo, Trey Gooch, Kelly Dunn, Florence Breslin, Julie M. Croff

**Affiliations:** Department of Psychiatry and Behavioral Sciences, Oklahoma State University Center for Health Sciences, Tulsa, OK, USA; and Director of Office of Medical Student Research, Office of Medical Student Research, Oklahoma State University College of Osteopathic Medicine at Cherokee Nation, Tahlequah, OK, USA; Oklahoma State University Center for Health Sciences, 1111 W 17th Street, Tulsa, OK 74107, USA; Office of Medical Student Research, Oklahoma State University College of Osteopathic Medicine at Cherokee Nation, Tahlequah, OK, USA; Office of Medical Student Research, Oklahoma State University College of Osteopathic Medicine at Cherokee Nation, Tahlequah, OK, USA; Department of Psychiatry and Behavioral Sciences, Oklahoma State University Center for Health Sciences, Tulsa, OK, USA; Department of Rural Health, Oklahoma State University Center for Health Sciences, Tulsa, OK, USA; National Center for Wellness and Recovery, Tulsa, OK, USA; and Professor, Department of Rural Health, Oklahoma State University Center for Health Sciences, Tulsa, OK, USA

**Keywords:** prenatal alcohol exposure, prenatal opioid exposure, prenatal substance exposure, prenatal tobacco exposure

## Abstract

**Context:**

Prenatal substance exposure (PSE) can lead to various harmful outcomes for the developing fetus and is linked to many emotional, behavioral, and cognitive difficulties later in life. Therefore, examination of the relationship between the development of associated brain structures and PSE is important for the development of more specific or new preventative methods.

**Objectives:**

Our study’s primary objective was to examine the relationship between the physical development of the amygdala, hippocampus, and parahippocampus following prenatal alcohol, tobacco, and prescription opioid exposure.

**Methods:**

We conducted a cross-sectional analysis of the Adolescent Brain and Cognitive Development (ABCD) Study, a longitudinal neuroimaging study that measures brain morphometry from childhood throughout adolescence. Data were collected from approximately 12,000 children (ages 9 and 10) and parents across 22 sites within the United States. Prenatal opioid, tobacco, and alcohol use was determined through parent self-report of use during pregnancy. We extracted variables assessing the volumetric size (mm^3^) of the amygdala, hippocampus, and parahippocampal gyrus as well as brain volume, poverty level, age, sex, and race/ethnicity for controls within our adjusted models. We reported sociodemographic characteristics of the sample overall and by children who had PSE. We calculated and reported the means of each of the specific brain regions by substance exposure. Finally, we constructed multivariable regression models to measure the associations between different PSE and the demographic characteristics, total brain volume, and volume of each brain structure.

**Results:**

Among the total sample, 24.6% had prenatal alcohol exposure, 13.6% had prenatal tobacco exposure, and 1.2% had prenatal opioid exposure. On average, those with prenatal tobacco exposure were found to have a statistically significant smaller parahippocampus.

**Conclusions:**

We found a significant association between prenatal tobacco exposure and smaller parahippocampal volume, which may have profound impacts on the livelihood of individuals including motor delays, poor cognitive and behavioral outcomes, and long-term health consequences. Given the cumulative neurodevelopmental effects associated with PSE, we recommend that healthcare providers increase screening rates, detection, and referrals for cessation. Additionally, we recommend that medical associations lobby policymakers to address upstream barriers to the effective identification of at-risk pregnant individuals, specifically, eliminating or significantly reducing punitive legal consequences stemming from state laws concerning prenatal substance use.

Prenatal substance exposure (PSE) can lead to various harmful outcomes for the developing fetus such as spontaneous abortion, structural malformations, growth retardation, central nervous system damage, and neurodevelopmental disorders [1]. According to the 2020 National Survey on Drug Use and Health (NSDUH), alcohol, tobacco/nicotine, and prescription pain relievers (opioids) are among the most frequently utilized substances [2]. These substances are also frequently utilized throughout pregnancy [3]. Despite there being no safe limit on alcohol consumption during pregnancy, one in seven pregnant individuals report current drinking (defined as having one alcoholic beverage in the last 30 days) during pregnancy, and 1 in 20 report binge drinking (defined as having consumed four or more drinks at least once in the last 30 days) [4]. Increased education and age have been associated with a higher likelihood of maternal alcohol consumption [5]. Additional substances, specifically tobacco smoking and prescription opioids, are also highly prevalent among pregnant people, with 6.6 % of pregnant individuals self-reporting prescription opioid use according to the 2019 Pregnancy Risk Assessment Monitoring System (PRAMS) Survey [6]. Among these individuals, 21.2 % reported misuse, utilizing for a reason other than pain, or receiving from someone other than a healthcare provider [6]. Because tobacco is the most prevalently utilized addictive substance worldwide, the rate of tobacco use among pregnant individuals was estimated to be 6.9 % in 2017 [7], and prenatal cigarette use has been found to have a dose-response association with all-cause and cause-specific infant death [8].

PSE has also been linked to memory deficits within children and may lead to emotional, behavioral, and cognitive difficulties later in life [9]. Opioid use during pregnancy has been shown to cause low birthweight (LBW) and symptoms of withdrawal in the infant, with observable changes in psychosocial patterns and cognitive and behavioral development [10]. Fetal tobacco exposure is also linked to LBW, intrauterine growth restrictions, altered attention levels, irritability, and hypertonicity during childhood [11]. Abnormalities of newborn neurobehavior, involving orientation and autonomic regulation, have also been identified in infants born with prenatal nicotine exposure [12]. These characteristics are found to continue through childhood, adolescence, and young adulthood, presenting as negative behavioral and cognitive outcomes, and may lead to delinquency, criminal behavior, and substance use during adulthood [13]. Fetal alcohol syndrome (FAS), the most severe form of fetal alcohol spectrum disorder (FASD), is considered to be one of the leading identifiable causes of intellectual disability, which is one example of an observable deficit in behavior and cognition [14]. Children with FAS have also been observed to be tremulous, hyperactive, and irritable, and have shown impairment in the ability to inhibit impulsive responses [15]. When children with FASD were evaluated utilizing the Behavior Rating Inventory of Executive Functioning questionnaire, they had the greatest difficulty with inhibitory control, working memory, and problem-solving [16].

Maternal alcohol consumption has been documented to affect behavior and cognition in infants as well as to alter brain structure and function [17]. A study evaluating the neurodevelopmental trajectory following prenatal alcohol exposure found that regarding cortical volume, thickness, and gyrification, individuals who were diagnosed with FASD had atypical cortical trajectories [17]. Other research has shown that total brain volume, as well as specific regions, are also likely to be affected by prenatal tobacco and opioid exposure [18]. Given the susceptibility of fetal neuroanatomy and the developmental impacts of PSE, focusing research on critical brain regions may help target approaches to improve the psychological and developmental outcomes of children. The amygdala receives and processes information relevant to emotion, cognition, and behavior, including fear and anxiety, punishment and reward, and decision-making [19]. Studies on alcohol consumption during pregnancy have shown altered development of nuclei in the amygdala, providing support for the susceptibility of this structure [20]. Furthermore, adults with alcohol use disorders were found to have smaller amygdalar volume in comparison to healthy controls [21].

Nicotine dependence among adults is also associated with altered amygdala function, and during abstinence, cravings interfered with amygdala response to harm signals [19]. In conjunction with the amygdala, the hippocampus and parahippocampus also help regulate socio-emotional functioning, learning, memory, and the neuroendocrine response to stress [22, 23]. Studies have found that individuals with larger alcohol use indexes had accelerated volumetric declines in the hippocampus and parahippocampus, which was associated with poorer memory performance [24]. Furthermore, nicotine exposure across the lifespan – including prenatal and adolescent – has been associated with unique behavioral, functional, and morphological outcomes for the hippocampus [25]. Opioid use has also been found to alter the functional connectivity for certain regions of the brain involved in emotion and cognitive and decision-making processes, including the hippocampus, parahippocampus, and amygdala [26].

For this study, we aimed to examine the relationship between the physical development of the amygdala, hippocampus, and parahippocampus following PSE (specific to the most frequently utilized substances: alcohol, tobacco, and prescription opioids) among 9- and 10-year-old children and those without PSE, because such observed structural changes may result in poor behavioral outcomes in children with PSE, which may require more specific or new treatment methods [27]. Our study will utilize baseline data from the Adolescent Brain and Cognitive Development (ABCD) Study, which has been utilized to investigate associations between PSE and early adolescent behavioral and cognitive abilities [28]. The ABCD Study is a large-scale, longitudinal open-data neuroimaging study that includes functional magnetic resonance imaging (fMRI) activation patterns and structural magnetic resonance imaging (sMRI) measures of brain morphometry, which were unavailable to researchers previously [29, 30].

## Methods

We performed a secondary analysis of data collected during the initial phase of the ABCD Study (https://abcdstudy.org/; data release 4.0.1). This study enrolled approximately 12,000 children born between 2006 and 2008 (ages 9 and 10) and parents across 22 sites within the United States. The study’s main purpose is to collect longitudinal magnetic resonance imaging (MRI) from child and adolescent participants throughout their adolescence, for at least 10 years. Parents and children were recruited through a probability sampling strategy of public and private schools, summer camp programs, community volunteers, birth registries, non–school-based community outreach, and word-of-mouth referrals. The inclusion criteria consisted of being in the required age range and the ability to provide informed consent (parents) and assent (child). The exclusion criteria included: lack of English language proficiency in the children; the presence of severe sensory, intellectual, medical, or neurological conditions that may impact the validity of the data; and a contra-indication to MRI scanning. We utilized the baseline data from release 4.0, representing eligible children from September 2016 to October 2018. Additionally, multiple surveys, cognitive assessments, and medical history were collected from or about the children, and medical history and sociodemographic items were collected from the parents or guardians. For this data release, 11,875 children had completed the phase with data available to be included in this analysis.

### Substance use

Prenatal opioid exposure was determined through the biological parent’s self-report of prescribed medication or illicit drug use. Within the medication history, prenatal opioid exposure was determined from questions asking about illicit Oxycontin or Morphine use of the biological mother during pregnancy, or if the mother had been prescribed any of the following drugs during pregnancy: Oxycontin, Oxycodone, Oxymorphone, Lortab, Percocet, Codeine, Vicodin, Morphine, Hydrocodone, Fentanyl, Norco, and Methadone. Prenatal alcohol and tobacco exposure was assessed through reported use during pregnancy – regardless whether the use was before or after the pregnancy and was known to the mother. Prenatal alcohol exposure was then split into three categories (none [0], 1–3, or 4+) based on alcohol consumption, measured as the average number of drinks per week. Tobacco use was coded as a binary variable as having utilized or not during pregnancy.

### Magnetic resonance imaging (MRI)

MRI within the ABCD Study was harmonized across 22 sites to allow for multi-site collaboration, as extensively discussed in a previous publication [31]. Quality control variables were utilized to exclude participants with indeterminable or corrupt MRI data. Then we extracted variables to assess the volumetric size (mm^3^) of the amygdala, hippocampus, and parahippocampal gyrus. The variable names within the dataset are *smri_vol_scs_amygdalalh* (left) and *smri_vol_scs_amygdalarh* (right) for the amygdala, *mrisdp_476* and *mrisdp_550* for the parahippocampal gyrus (Destrieux parcellation) [32], and *smri_vol_scs_hpuslh* and *smri_vol_scs_hpusrh* for the hippocampus, whereas total brain volume, utilized as a control for these analyses, was assessed utilizing the variable *smri_vol_scs_wholeb*.

### Demographics

Volumetric differences in both the amygdalar and hippocampal regions in children have been noted on poverty level [33] and among adults and nonhuman animals who were exposed to chronic stress. Given these differences, we extracted data regarding household financial stability to assess the financial risk – a cumulative measure of seven items assessed as the number of affirmative responses to the following prompts: *In the past 12 months*, *has there been a time when you and your immediate family experienced any of the following*:

1. Needed food but couldn’t afford to buy it or couldn’t afford to go out and get it?
2. Were without telephone service because you could not afford it?
3. Didn’t pay the full amount of the rent or mortgage because you could not afford it?
4. Were evicted from your home for not paying the rent or mortgage?
5. Had services turned off by the gas or electric company, or the oil company wouldn’t deliver oil because payments were not made?
6. Had someone who needed to see a doctor or go to the hospital but didn’t because you could not afford it?
7. Had someone who needed a dentist but couldn’t go because you could not afford it?

Additionally, the interaction of age, sex assigned at birth, and race/ethnicity could influence variations in growth. Therefore, we utilized these variables, along with brain volume (mentioned previously) and financial risk as controls within the adjusted models. Age (in months at the time of the survey), sex, and race/ethnicity were are collected by parent report.

### Statistical analysis

We applied the complex design of the survey data to stratify for recruitment sites and family units. We reported the sociodemographic characteristics of the sample overall and by children who were prenatally exposed to alcohol, tobacco, or prenatal opioids. Next, we calculated and reported the means of each brain region by substance exposure type utilizing the sum of the left and right measures for each of the brain structures – amygdala, parahippocampal gyrus, and hippocampus. We then constructed multivariable regression models to measure the associations between different PSE, controlling each of the other types, as well as the demographic characteristics described previously, total brain volume, and the volumes of each brain structure. Data collection for the ABCD Study was approved by the central Institutional Review Board (cIRB) at the University of California, San Diego, CA, USA. Results were reported according to Strengthening the Reporting of Observational Studies in Epidemiology (STROBE) guidelines.

## Results

The number of ABCD Study child participants was 11,755 – with descriptive statistics of children with PSE presented in Table 1. Of the total sample, 24.6 % (n=2,892) had prenatal alcohol exposure, 13.6 % (n=1,604) had prenatal tobacco exposure, and 1.2 % (n=149) had prenatal opioid exposure, while 67.8 % (n=7,977) had no exposure to any of these substances. The mean age of the sample was 9.9 years (SD=0.6), with little difference observed between exposure groups. In terms of sex assigned at birth, 52.2 % (n=6,140) were female, and 47.7 % (n=5,615) were male, with similar exposure percentages for both groups. Regarding race/ethnicity, a majority (52.1 %, n=6,133) reported as White, followed by 20.3 % (n=2,395) Hispanic, 14.7 % (n=1,739) Black, and 10.5 % (n=1,239) other races. The mean financial risk was higher in the tobacco (mean [M]=0.9; standard deviation [SD]=1.5) and opioid exposure (M=0.9; SD=1.4) groups than in the other groups. Total brain volume was similar across exposure groups.

The average volumetric size for the brain structures are reported in Table 2. Our results showed that children with prenatal alcohol exposure on average have smaller amygdala and hippocampus when compared to children without prenatal alcohol exposure. Those with prenatal opioid exposure had smaller amygdala and parahippocampus volumes while having a larger hippocampus, compared to children with no prenatal opioid exposure. However, alcohol and opioid exposure were not statistically significant differences.

From our regression analysis, the only significant relationship with prenatal exposure was between prenatal tobacco use and the parahippocampus – with those exposed having a −212.1 mm^3^ (SD=32.2) smaller region on average compared to those without prenatal tobacco exposure, while controlling for the other variables in the model (*z*=−6.5, p<0.001; Table 2). However, there were significant associations between demographic variables utilized as controls and volumes of the amygdala, parahippocampus, and hippocampus – including race and sex assigned at birth.

## Discussion

Our study found that children with prenatal tobacco exposure on average had a smaller parahippocampal volume – an association that has been identified in previous studies [34]. Among the other variables tested, our results showed little deviation in volumetric sizes of the amygdala and parahippocampus among those with prenatal exposures to alcohol, tobacco, or opioid use. While our results showed little deviation in these specific brain regions among these 9- and 10-year-old children, there is a plethora of research showing the adverse effects of prenatal exposures to alcohol, tobacco, and opioids among infants and younger children [35]. Given the lifelong plasticity found in this brain region, these brain structures may have the ability to alter their growth patterns, thereby regaining a more normal size by this age [36].

Because we found that the significant relationship between tobacco exposure and reduced parahippocampal volume was still present within our sample at 9 and 10 years of age, and because the parahippocampus is linked to memory encoding and retrieval [37], there may be longer-term implications that may result in cognitive delay or lower performance in traditional US school systems. Further, this area of the brain is linked to complex emotion regulation – specifically negative response patterns, which may be linked to attention deficit hyperactivity disorder (ADHD), anxiety, and panic disorders, as well as other child and adolescent behavioral issues [38]. These issues specific to the parahippocampus are in addition to the known effects of prenatal nicotine exposure including poorer maternal and childbirth outcomes, early motor delays, poorer spatial orientation, and long-term health consequences [39]. However, regarding the parahippocampus, a previous study showed that cognitive behavioral therapy (CBT) was effective in improving symptoms of anxiety and panic disorders through reduced parahippocampal activation.

### Prevention

Given the cumulative neurodevelopmental effects associated with PSE, risk assessment for substance use among pregnant individuals is increasingly important because they are at increased risk for adverse health and social outcomes [40]. Research indicates that a substantial number of pregnant women cared for during the prenatal period have unrecognized or untreated substance use patterns [41]. Tools for identifying prenatal substance use outside of biological sample testing include validated screening tools such as the 4 P’s Plus (parents, partner, past, and pregnancy substance use screen) [42], National Institute on Drug Abuse (NIDA) Quick Screen-ASSIST (Modified Alcohol, Smoking, and Substance Involvement Screening Test), and the SURP-P (Substance Use Risk Profile-Pregnancy) scales. Although the 4 P’s Plus and the NIDA Quick Screen-ASSIST [43] have been shown to have high sensitivity and negative predictive values, a 2010 survey of obstetrician-gynecologists (OB/GYNs) found 58 % did not utilize a validated screening tool to assess alcohol risk despite there being several validated tools available [44]. This may relate to both tobacco screening and opioid use.

In addition to identifying substance use, the intervention methods following identification play an important role in patient outcomes. Studies have found that many women recognize that there are risks associated with drinking during pregnancy, but they do not receive individual advice from health professionals – revealing a lack of clear and consistent advice regarding safe drinking levels from health professionals [45]. Primary care studies have provided evidence that physician intervention and counseling through advice, education, and contracting information significantly reduces the amount of alcohol use, episodes of binge drinking, and frequency of excessive drinking [46]. However, there may be less motivation or perceived barriers for healthcare workers to implement interventions after screening – especially for tobacco use. Among a 2014 study of 252 OB/GYNs, 88 % reported always asking about tobacco use during an initial visit; however, the sample also reported that only 25 % asked about smoking status if it was suspected but not marked on the patient intake, which may under-report smoking status. Further, if tobacco use was identified, the most frequent action was to advise abstinence and the least frequent was to refer to counseling [47], despite the United States Preventive Services Task Force (USPSTF) advising to “provide behavioral interventions for cessation” in addition to advising to stop [48]. The respondents in the study reported the most perceived barriers to offering intervention being a lack of referral resources for prenatal tobacco use, expecting patient denial or treatment resistance, and most commonly, time limitations [47].

### Recommendations

While our first recommendation is for healthcare providers to increase screening rates, detection, and referrals for cessation, also noted by Zou et al. [49], we also recommend that medical associations lobby policymakers to address upstream barriers to the effective identification of at-risk pregnant individuals, specifically eliminating or significantly reducing punitive legal consequences stemming from state laws concerning prenatal substance use – particularly from opioids and other controlled drugs [50, 51]. These laws discourage disclosure of substance use behaviors to providers and ultimately delay intervention and treatment – which also disproportionately affects individuals with low socioeconomic status and people of color [52]. Further, these laws also propagate public stigma surrounding substance use among pregnant individuals. A qualitative study of individuals who utilized substances during pregnancy not only highlighted this stigma to the point of self-isolation and avoidance of seeking care, but it also highlighted the lack of knowledge of treatment options for them and the gaps in services after pregnancy [52]. For instance, among a group of mothers who were on methadone during pregnancy, they often reported having long periods after pregnancy when they were not able to continue treatment and had desires to resume drug use [52]. Thus, increasing both the public messaging surrounding substance use and postnatal services may reduce the long-term impact of prenatal substance use and therefore outcomes from prenatal exposure.

A 2022 study evaluating the consumption of alcohol during pregnancy found that some of the most commonly reported reasons for alcohol use during pregnancy were due to the lack of awareness of adverse effects for the fetus, a belief that only “strong” alcohol or alcohol in large quantities would be harmful, and advice from medical practitioners [53]. Additionally, a study evaluating tobacco use in pregnancy identified risk factors such as a lower education status, clinically relevant anxiety symptoms, and exposure to environmental tobacco smoke [54]. This information further supports the previously mentioned need for physician intervention and counseling through advice, education, and contracting information regarding alcohol and tobacco use in pregnancy. Opioid use during pregnancy is due to a variety of reasons, and commonly is due to the pain associated with pregnancy – with the prevalence of low back and pelvic pain during pregnancy ranging from 68 to 72 % [55]. Osteopathic manipulative treatments (OMTs) prove to be an effective tool to reduce the acute pain of the lower back and pelvis during pregnancy [56]. Additionally, these treatments have also been found to improve the mental health and emotional aspects of the patients receiving treatments [56]. Therefore, osteopathic manipulative medicine (OMM) may provide an approach to care for pregnant individuals that can reduce both opioid and tobacco use during pregnancy.

### Limitations

One of the limitations of the ABCD Study was that maternal medical history was performed via self-survey by the patient rather than collected from a medical chart, leading to subjectivity in the responses, and it may also be subject to recall bias given that the surveys were collected 9–10 years following the pregnancy. The use of prescription opioids may also have been underreported during the height of the opioid pandemic. Along these lines, the report of substance use during pregnancy, or even right after pregnancy, may have been skewed due to the stigma surrounding tobacco, alcohol, and opioid use during pregnancy. It is worth noting that substance use groupings may have overlap between columns – some individuals may be exposed to more than one substance – and therefore caution should be taken when interpreting the data. It is also important to note that this study is conducted on children ages 9 and 10, which may not yield the most precise results regarding prenatal exposure due to the inherent plasticity of the infant/pediatric brain. However, given the large sample size and robust methodology within the ABCD Study across all 23 sites, we believe that these limitations are minimized. Given the previous research linking MRI measurements of the parahippocampal gyrus-orbitofrontal cortex to memory loss and cognitive impairment among individuals with Alzheimer’s disease, a future cohort study to investigate the relationship between changes in the brain structure of individuals who were exposed to prenatal tobacco and their likelihood of developing Alzheimer’s disease may be warranted. Another research initiative, The HEALthy Brain and Child Development Study, may be another avenue to investigate linkages between PSE and infant brain development, as this study is following cohorts of pregnant women to better understand substance use, PSE, and pre- and postnatal outcomes [57].

## Conclusions

Fetal brain development is sensitive to foreign substances and thus can be altered when exposed to potentially harmful substances. Our results showed a significant association between prenatal tobacco exposure and smaller parahippocampal volume, which may have profound impacts on the livelihood of these individuals, including motor delays, poor cognitive and behavioral outcomes, and long-term health consequences. For alcohol and opioid exposure, we found no significant difference in structure volumes – which may be due to the plasticity of the region. Future research should track earlier brain development of this region to assess its adaptability.

## Figures and Tables

**Table 1:** Descriptive statistics of children with PSE in the ABCD study (n=11,755).

	Prenatal alcohol use 2,892 (24.6 %)	Prenatal tobacco use 1,604 (13.6 %)	Prenatal opioid (Rx) use 149 (1.2 %)	No exposure (to these 3) 7,977 (67.8 %)	Total 11,755 (100 %)
Age, years					
Mean (SD)	9.91 (0.63)	9.94 (0.63)	9.92 (0.66)	9.91 (0.62)	9.91 (0.62)
Sex at birth (No. and % with exposure)					
Female	1,478 (24.0)	842 (13.7)	79 (1.2)	4,184 (68.1)	6,140 (52.2)
Male	1,414 (25.1)	762 (13.5)	67 (1.1)	3,790 (67.5)	5,615 (47.7)
Race/Ethnicity (No. and % with exposure)					
White	1,816 (29.6)	747 (12.1)	77 (1.2)	3,953 (64.4)	6,133 (52.1)
Black	271 (15.5)	326 (18.7)	17 (0.9)	1,245 (71.5)	1,739 (14.7)
Hispanic	470 (19.6)	279 (11.6)	29 (1.2)	1,772 (73.9)	2,395 (20.3)
Asian	35 (14.0)	9 (3.6)	0 (0)	213 (85.2)	250 (2.1)
Other races not listed	299 (24.1)	243 (19.6)	23 (1.8)	793 (64.0)	1,239 (10.5)
Financial risk (household)					
M (SD)	0.44 (1.08)	0.99 (1.5)	0.92 (1.45)	0.41 (1.02)	0.47 (1.1)
Total brain volume, mm^3^					
M (SD)	1,236,602 (114,137.8)	1,206,039 (111,593)	1,208,033 (121,728.1)	1,218,784 (113,147.8)	1,221,941 (113,894.5)

Substance use groupings may have overlap between columns. ABCD, adolescent brain and cognitive development; M, mean; PSE, prenatal substance exposure; SD, standard deviation.

**Table 2:** Differences in the volume of the amygdala, parahippocampal gyrus, and hippocampus, by prenatal exposure and associations.

Variable	Amygdala		Parahippocampus		Hippocampus	
	Mean (SD) mm^3^	Adjusted model Coef (SE)	Mean (SD) mm^3^	Adjusted model Coef (SE)	Mean (SD) mm^3^	Adjusted model Coef (SE)
Alcohol consumption (drinks per week)						
None (0)	3,534.23 (421.74)	1 (reference)	7,277.33 (1,340.76)	1 (reference)	8,143.56 (790.13)	1 (reference)
>0–3	3,571.03 (442.89)	−10.12 (9.13)	7,414.58 (1,369.41)	34.55 (35.84)	8,236.62 (811.11)	−10.79 (14.66)
4+	3,587.16 (430.19)	−8.9 (12.31)	7,403.73 (1,429.95)	25.12 (55.13)	8,267.97 (777.00)	−12.18 (23.35)
Prenatal tobacco use						
No	3,547.53 (429.55)	1 (reference)	7,334.83 (1,357.69)	1 (reference)	8,174.14 (798.03)	1 (reference)
Yes	3,488.72 (401.52)	−10.47 (8.72)	7,051.14 (1,283.2)	−212.15 (32.26)	8,056.80 (774.21)	−17.42 (14.92)
Prenatal rx opioid use						
No	3,540.30 (425.79)	1 (reference)	7,297.43 (1,349.47)	1 (reference)	8,158.78 (795.35)	1 (reference)
Yes	3,476.03 (461.81)	−14.91 (35.28)	7,192.82 (1,484.8)	−104.01 (149.38)	8,107.10 (832.22)	36.76 (55.69)
Race/ethnicity						
White	3,623.42 (426.19)	1 (reference)	7,532.37 (1,375.18)	1 (reference)	8,329.80 (765.24)	1 (reference)
Black	3,350.70 (390.45)	−38.07 (9.23)^b^	6,951.74 (1,203.82)	−136.69 (35.59)	7,737.19 (772.59)	−78.7 (19.03)
Hispanic	3,494.77 (409.97)	−4.84 (7.83)	7,049.02 (1,288.19)	−258.43 (34.79)	8,057.47 (773.77)	−4.27 (12.31)
Asian	3,485.34 (375.53)	−45.48 (21.98)	6,665.95 (1,187.27)	−707.72 (78.60)	8,143.04 (699.70)	54.93 (42.86)
Race not listed	3,486.19 (406.82)	−63.11 (10.97)^b^	7,212.07 (1,336.25)	−171.16 (41.97)	8,095.78 (771.26)	−69.45 (16.61)
Sex at birth						
Female	3,687.98 (416.32)	1 (reference)	7,477.17 (1,428.34)	1 (reference)	8,416.96 (770.34)	1 (reference)
Male	3,376.94 (374.40)	−69.59 (7.01)^b^	7,098.09 (1,231.76)	70.65 (2,752)	7,874.96 (723.58)	−14.12 (12.31)

Adjusted models also controlled for interview age (in months), financial risk score, and whole brain volume.

a p<0.01

b p<0.01.

Coef, coefficient; SD, standard deviation; SE, standard error.

## Data Availability

Not applicable.
